# An Efficient Estimator of the Mutation Parameter and Analysis of Polymorphism from the 1000 Genomes Project

**DOI:** 10.3390/genes5030561

**Published:** 2014-07-22

**Authors:** Yunxin Fu

**Affiliations:** 1 Division of Biostatistics and Human Genetics Center, The University of Texas Health Science Center at Houston, 1200 Herman Pressler, Houston, TX 77025, USA; E-Mail: yunxin.fu@uth.tmc.edu; Tel.: +1-713-500-9813; Fax: +1-713-500-0900; 2 Laboratory for Conservation and Utilization of Bio-Resources, Yunnan University, Kunming 650091, China

**Keywords:** mutation parameter, coalescent, allelic genealogy, 1000 Genomes Project

## Abstract

The mutation parameter *θ* is fundamental and ubiquitous in the analysis of population samples of DNA sequences. This paper presents a new highly efficient estimator of *θ* by utilizing the phylogenetic information among distinct alleles in a sample of DNA sequences. The new estimator, called Allelic BLUE, is derived from a generalized linear model about the mutations in the allelic genealogy. This estimator is not only highly accurate, but also computational efficient, which makes it particularly useful for estimating *θ* for large samples, as well as for a large number of cases, such as the situation of analyzing sequence data from a large genome project, such as the 1000 Genomes Project. Simulation shows that Allelic BLUE is nearly unbiased, with variance nearly as small as the minimum achievable variance, and in many situations, it can be hundreds- or thousands-fold more efficient than a previous method, which was already quite efficient compared to other approaches. One useful feature of the new estimator is its applicability to collections of distinct alleles without detailed frequencies. The utility of the new estimator is demonstrated by analyzing the pattern of *θ* in the data from the 1000 Genomes Project.

## 1. Introduction 

The pattern of genetic polymorphism in a population can be influenced by a number of factors, among which the mutation parameter (commonly denoted by *θ*) plays a central role. *θ* is defined as 4*Nu* and 2*Nu* for diploid and haploid genomes, respectively, where *N* is the effective population size and *u* is the mutation rate per sequence per generation. Almost all existing summary statistics for polymorphism are related to *θ*. Well-known examples include the number of alleles in a sample [1] the number (K) of segregating sites (or polymorphic sites) [2], mean number (*Π*) of nucleotide differences between two sequences [3] and the number of mutations of various sizes [4]. 

Due to the fundamental nature of this parameter for understanding both population dynamics, as well as the mechanism of evolution, it is important that it can be estimated as accurately as possible. Classical estimators include Watterson’s estimator [2], Tajima’s estimator [3], Ewens’ estimator based on the number of alleles [5] in the sample and the heterozygosity estimator [6]. Under the assumption of a single random mating population evolving according to the Wright–Fisher model with constant population size and neutral mutations, these estimators are all either unbiased or nearly unbiased. However, their variances, which are the primary measure of accuracy of an estimator, can differ considerably and, furthermore, are substantially larger than the minimum achievable variance [7]:

(1) $$V_{\min}=\theta {\left[\sum_{i=1}^{n-1}\frac{1}{\theta +i}\right]}^{-1}$$

where *n* is the sample size. Realizing the limitations of these classical estimators, several new approaches were developed in the 1990s, all utilizing the fine structural result of coalescent theory [3,8,9]. Representative are Griffiths and Tavare’s Markov Chain Monte Carlo (MCMC) estimator [10,11] based on recurrent equations for the probability of the polymorphism configuration, Knuher and Felsenstein’s MCMC method [12] based on Metropolitan-Hasting sampling and Fu’s BLUE estimators [13,14] based on linear regression taking advantage of the linear relationship between mutations in the genealogy of a sample and the mutation parameter. These new groups of estimators can all achieve substantially smaller variances and may even reach the minimum variance [13]. One common feature of these estimators is that they are all computationally intensive and, as a result, are suitable for only relatively smaller samples. Such limitations are particularly serious for the MCMC-based approach. 

The potential for genetic research based on population samples has been greatly enhanced by the steady reduction in the cost of sequencing. As a result, sample sizes in these studies are substantially larger than before, and the trend will continue with the arrival of next generation sequencers. Already, it is commonplace to see sequenced samples of many hundreds of individuals and even thousands (such as the sample in the 1000 Genomes Project [15]). The reduction of sequencing cost also leads to a larger region of the genome or even the entire genome being sequenced (e.g., 1000 Genomes Project). Consequently, new approaches that are both highly accurate and efficient in computation are desirable. This paper presents one such method and demonstrates its utility by analyzing polymorphism from the 1000 Genomes Project. 

## 2. Theory and Method 

### 2.1. The Theory 

**Figure 1:**
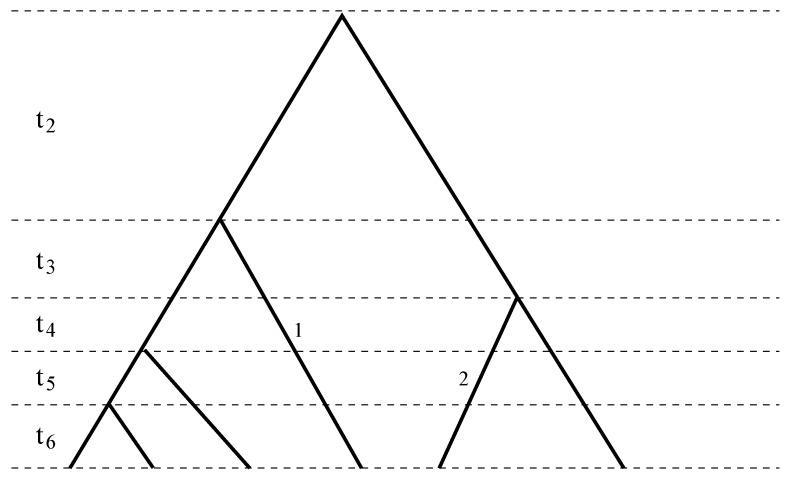
A sample genealogy with different coalescent times separated by dashed lines. Branch 1 spans the third to the sixth coalescent times, *χ*_1_(2) = 0, *χ*_1_(*i*) = 1, for *i* = 3, ..., 6, while Branch 2 spans the fourth to the sixth coalescent times, *χ*_2_(2) = *χ*_2_(3) = 0, *χ*_2_(4) = *χ*_2_(5) = *χ*_2_(6) = 1. Combining Branches 1 and 2 results in *ϕ*(2) = 0, *ϕ*(3) = 1, *ϕ*(4) = *ϕ*(5) = *ϕ*(6) = 2.

Assume that a sample of *n* DNA sequences at a locus without recombination is taken from a single population evolving according to the Wright–Fisher model and all mutations are selectively neutral. The sample genealogy thus consists of 2(*n* − 1) branches, each spanning at least one coalescent time (Figure 1). The number of mutations that occurred in a branch is thus the sum of the numbers of mutations in the coalescent time it spans. Consider one branch, and without loss of generality, assume it spans the i-th coalescent time. Then, during the i-th coalescent time, the number of mutations occurred in the branch has expectation and variance equal to:

(2) $$\frac{\theta }{i(i-1)}\text{ and }\frac{\theta }{i(i-1)}+\frac{1}{i^{2}{\left(i-1\right)}^{2}}\theta^{2}$$

respectively. These are consequences of the coalescent time being exponentially distributed and the number of mutations in a given number of generations following a Poisson distribution. Consider the number of mutations in another branch that spans the j-th coalescent time. Then, the covariance between the two numbers of mutations is equal to:

(3) $$\frac{\theta^{2}}{i^{2}{\left(i-1\right)}^{2}}$$

if *i* = *j*, and zero otherwise. 

For the branch *k*(*k* = 1, ..., 2(*n* − 1)) in the genealogy, define an index *χ_k_*(*i*), such that it takes value one if the branch spans the *i-*th coalescent time and zero otherwise. Then, *m_k_*, the number of mutations, has its expectation and variance equal to:

(4) $$E(m_{k})=\theta \sum_{i=2}^{n}\frac{\chi_{k}(i)}{i(i-1)}$$

(5) $$V(m_{k})=E(m_{k})+\theta^{2}\sum_{i=2}^{n}\frac{\chi_{k}^{2}(i)}{i^{2}{\left(i-1\right)}^{2}}$$

respectively, and for two different branches *a* and *b*:

(6) $$Cov(m_{a},m_{b})=\theta^{2}\sum_{i=2}^{n}\frac{\chi_{a}(i)\chi_{b}(i)}{i^{2}{\left(i-1\right)}^{2}}$$

The previous results are readily generalized. Instead of considering the mutations in different branches separately, one can combine mutations in several branches. Suppose branches (*k*_1_, ..., *k_t_*) are combined. Define for the combined branches a variable *ϕ* as:

(7) $$\phi (i)=\sum_{j=1}^{t}\chi k_{j}(i)$$

Consider a population dynamics model in which the effective population size can change only at the time a coalescent event occurs. Although such a model does not stem from biological reality, its laddered changes in population sizes allow a reasonable approximation of reality and makes the mathematics simpler. Let *θ_i_* represent the *θ* during the i-th coalescent period. Suppose the combined branches is denoted by branch (group) *k*, then *m_k_*, the number of mutations in branch k has expectation and variance equal to:

(8) $$E(m_{k})=\sum_{i=2}^{n}\frac{\phi_{k}(i)\theta_{i}}{i(i-1)}$$

(9) $$V(m_{k})=E(m_{k})+\sum_{i=2}^{n}\frac{\phi_{k}^{2}(i)\theta_{i}^{2}}{i^{2}{\left(i-1\right)}^{2}}$$

respectively, and for two such branches *a* and *b*, we have:

(10) $$Cov(m_{a},m_{b})=\sum_{i=2}^{n}\frac{\phi_{a}(i)\phi_{b}(i)\theta_{i}^{2}}{i^{2}{\left(i-1\right)}^{2}}$$

Suppose that the 2(*n* − 1) branches of the sample genealogy are divided into *M* (≤ 2(*n* − 1)) disjoint groups (*i.e.*, each branch belongs to one and only one group). Let *m_k_* represent the number of mutations in branch group *k* and *m* = (*m*_1_, ..., *m_k_*)*^T^*. Then, similar to the previous result [13], the relationship between *θ* = (*θ*_2_, ..., *θ_n_*)*^T^* and *m* can be expressed by a generalized linear model:

(11) *m* = *αθ* + *e*

where *α* is a matrix of dimension *M* × *n* with: $\alpha_{ij}=\frac{\phi_{i}(j)}{j(j-1)}$ and *e* a vector of length *M* representing error terms. Let Γ(*θ*)= *Var*(*m*). Then:

(12) Γ(*θ*) = *γ*(*θ*) + *β*(*θ*)

where *γ* and *β* are both *M* × *M* matrices defined as:

(13) *γ*(*θ*) = *Diag*{*α*_1_*θ*, ..., *α_M_θ*}

(14) $$\beta (\theta )=\left\{\sum_{k=2}^{n}\frac{\phi_{i}(k)\phi_{j}(k)\theta_{k}^{2}}{k^{2}{\left(k-1\right)}^{2}}\right\}$$

where *α_k_* represents the k-th row vector of *α*. Following the previous approach [13,14], a best linear unbiased estimator of *θ* can be obtained as the limit of the series:

(15) $$\theta^{\left(k+1\right)}={\left[\alpha^{T}\text{Γ}{\left(\theta^{\left(k\right)}\right)}^{-1}\alpha \right]}^{-1}\alpha^{T}\Gamma {\left(\theta^{\left(k\right)}\right)}^{-1}m$$

with *θ*^(0)^ being the initial estimate of ***θ*** (for example, setting all *θ_i_* equal to Watterson’s estimate of *θ*). 

The above formulation allows maximal *n* − 1 different values of *θ* corresponding to the *n* − 1 coalescent periods. Although very flexible, such an extreme model may lead to reduced accuracy of estimation for individual parameters, so some compromise is likely to be useful. When two or more consecutive *θ* values are assumed to be the same, the length of the *θ* vector will be reduced. At the extreme, if all of the *θ*s are the same, *θ* is reduced to a single quantity, and when *M* = 2 (*n* − 1), it further defaults to BLUE [13]. Since efficient estimators for a single value of *θ* will continue to be useful in the analysis of the whole genome polymorphism of large samples, we will focus on developing one such scheme in this paper. 

### 2.2. Allelic BLUE estimator 

In order to take advantage of the BLUE estimator, sample genealogy needs to be inferred. Furthermore, the key to developing a highly efficient BLUE estimator is to define the M groups of branches, which not only retains the detail mutational information, but also satisfies the relationship *M* << 2(*n* − 1). Fu’s UPBLUE [13] corresponds to the extreme in which *M* = 2(*n* − 1), *i.e.*, each branch belongs to its own group. While this may retain the maximal mutational information, it leads to computational inefficiency. Fu’s [14] approach is more or less equivalent to *M* = *n* − 1, with groups defined by the size of mutations. This achieves computational efficiency with the expense of reduced accuracy due to over condensation of mutational information. Thus, the goal here is to strive for a balance. 

**Figure 2:**
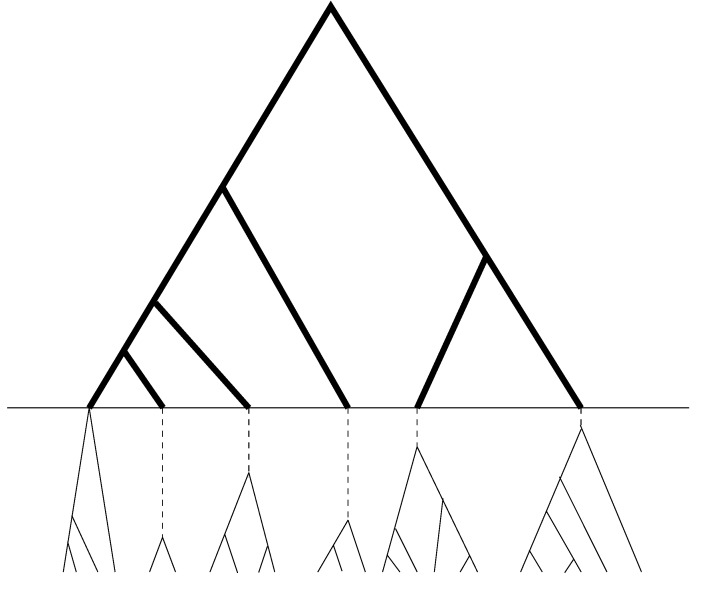
Schematic relationship between sample genealogy and allelic genealogy. The dark portion is the genealogy of distinct alleles, while the light portion (which is below the horizontal line) is the coalescent within alleles and contains no mutation or only a few in the dashed segments of the branches.

We recognize that much of the phylogenetic information in a sample resides in the pattern of differences between distinct alleles. The phylogenetic method, UPGMA (e.g., [16]), which was found to be appropriate in Fu [13], will continue to be used in our new method. Since UPGMA is a sequential method, which at each step joins two sequences (or two groups of sequences) that differ the least. As a result, copies of sequences of the same allele will be joined together before any pair of sequences of distinct alleles. The resulting sample genealogy can be roughly divided into two portions (see Figure 2), with the bottom portion corresponding to the coalescent within allelic class and the upper portion the coalescent among allelic classes. Combine all the branches (or segments of branches) underneath the dashed line into one group, which will be referred to as the within allele branch. Suppose there are *L* distinct alleles in the sample; then we have for the within allele branch:

(16) $$\phi (i)=\left\{ \begin{array}{l} 0,\;i\geq L\;\\ i,\;i>L \end{array}\right.$$

Then, the number of mutations in the within allele branch have expectation and variance equal respectively to:

(17) (*a_n_* − *a_L_*)*θ* and (*a_n_* − *a_L_*)*θ* + (*b_n_* − *b_L_*)*θ*^2 ^

Furthermore, we assume that there is no mutation in the within allele branch (which should be a good approximation, although technically, the assertion may not be true). Since the within allele branch does not span any coalescent time that overlaps with those of branches in the allelic genealogy, we have (assumed that the last branch group represents the within allele subtree) that:

(18) $$\alpha =\left(\begin{array}{l} \alpha^{*}\\ \alpha_{n}-\alpha_{L} \end{array}\right)$$

where *α*^∗^ a vector of length 2(*L* − 1) with the *k*-th element equal to $\sum_{i=2}^{L}\frac{\phi_{k}(i)}{i(i-1)}$. The inverse of the covariance matrix of *m* is:

(19) $$\left(\begin{array}{cc} {\text{Γ}}^{*}{\left(\theta \right)}^{-1} & 0\\ 0 & {\left[(a_{n}-a_{L})\theta +(b_{n}-b_{L})\theta^{2}\right]}^{-1} \end{array}\right)$$

where Γ^∗^ is defined for branch groups of the allelic genealogy. Let m be the vector of mutations in branches of the allelic genealogy (the dark portion in the genealogy of Figure 2). Then, Equation (15) becomes:

(20) $$\theta^{\left(k+1\right)}=\frac{{\left(\alpha^{*}\right)}^{T}{\text{Γ}}^{*}{\left(\theta^{\left(k\right)}\right)}^{-1}}{{\left(a_{n}-a_{L}\right)}^{2}{\left[(a_{n}-a_{L})+(b_{n}-b_{L})\theta^{\left(k\right)}\right]}^{-1}+{\left(\alpha^{*}\right)}^{T}{\text{Γ}}^{*}{\left(\theta^{\left(k\right)}\right)}^{-1}\alpha^{*}}m^{*}$$

This limit will be referred to as the Allelic BLUE estimator denoted by *θ_ab_*. Since, for large samples, the number of distinct alleles is typically much smaller than the sample size; thus, the new estimator is expected to be highly efficient computationally. 

To determine if it is indeed true that merging those branches representing within allele coalescent does not lead to significant loss of information and, thus, would not reduce the accuracy of estimation, we compared Allelic BLUE with the original BLUE using simulated samples for a number of combinations of *θ* and *n*. The correlation between the two estimates is around 0.99. Therefore, Allelic BLUE is expected to be as accurate as BLUE without merging branches. 

### 2.3. Bias-Corrected Allelic BLUE Estimator 

Since UPGMA systematically introduces bias in the inferred sample genealogy, the resulting Allelic BLUE estimate is expected to be biased similar to the BLUE estimator [13]. Therefore, it is necessary to correct the bias. Similar to Fu [13], we used simulated samples to derive understanding of the pattern of biases. A total of 550 combinations of *θ* and *n* were examined with 25 different *θ* values: 0.5, 0.75, 1, 1.5, 2(1)5, 6(2)12, 15(5), 50, 60(10)100 and 150, and 25 different sample sizes *n*: 10(5)25, 30(10)60, 80, 100(25)200, 250, 300, 400, 500, 750, 1000(1000)5000. For each combination of the parameters, 1000 samples were simulated, and for each simulated sample, *θ_ab_* was obtained and their mean value computed over all simulated samples. Similar to those in Fu [13], the estimates in almost all situations are underestimates of the true *θ*. In general, the underestimate is the result of systematic bias of the UPGMA algorithm used to construct the genealogy, because UPGMA leads to early coalescent for more similar sequences and, thus, has a tendency to place more mutations in branches that are deeper into the tree. In the current situation, it is further compounded by our simplification that, up to the *i* + 1 coalescent, there are no mutations. 

Using regression analysis, Fu [13] showed that the relationship:

(21) $$\sqrt{\theta_{u}}=-0.0336\sqrt{n-2}+1.002\sqrt{\theta }$$

summarizes well the BLUE estimate (with *M* = 2(*n* − 1)), *θ* and sample size n, which is not larger than 100. When larger sample sizes were examined, the above equation is not adequate, and log transformation, rather than square-root transformation, can lead to a better regression [17]. Therefore, log-transformation was chosen in our regression analysis. Table 1 showed that *ln*(*θ_ab_*) can be summarized very well by the following equation:

(22) *ln*(*θ_ab_*) = −0.1981 + 0.0080*ln*(*n*) + 1.0043*ln*(*θ*) − 0.0019*ln*(*n*)*ln*(*θ*) + 0.0108*ln*^2^(*θ*) − 0.0012*ln*(*n*)*ln*^2^(*θ*)

which leads to an estimate of *θ* from the solution for the above quadratic equation with regard to *ln*(*θ_a_*):

(23) $${\hat{\theta }}_{a}=\text{exp}\left[\frac{-b+\sqrt{b^{2}-4ac}}{2a}\right]$$

where:

(24) *a* = 0.0108 − 0.0012*ln*(*n*)

(25) *b* = 1.0043 − 0.0019*ln*(*n*)

(26) *c* = −*ln*(*θ_ab_*) − 0.1981 + 0.008*ln*(*n*)

Although this estimator in most situations is excellent, we found that regression equations for a narrower range of sample sizes provides estimates that are more robust in some situations (particularly when *θ* is large). As a result, we derive our final estimator ${\hat{\theta }}_{a}$ of *θ* using Equation (23) with values of *a*, *b* and *c*, as provided in Table 2. 

**Table 1:** Summary of regression analysis between *θ_ab_*, *θ* and *n*.

Source	Sum of Squares	Degrees of freedom	Mean Square	
Model	1,715.227	5	343.0454	
Residual	0.074	644	0.0001	
Total	1,715.301	649	2.6430	
**Term**	**Coef** **fi** **cient**	**Standard Error**	***t*** **test**	***P*** ** > ** **|** ***t*** **|**
*ln*(*n*)	0.0080	0.0005	16.73	0.000
*ln*(*θ*)	1.0043	0.0025	398.16	0.000
*ln*(*n*)*ln*(*θ*)	-0.0019	0.0004	-4.19	0.000
*ln*^2^(*θ*)	0.0108	0.0005	19.80	0.000
*ln*(*n*)*ln*^2^(*θ*)	-0.0012	0.0001	-12.74	0.000
constant	-0.1981	0.0027	-72.99	0.000

Note: Number of points for regression = 650, *R*^2 ^= 1.000 and MSE = 0.0107

**Table 2:** Coefficients for estimating *θ* using Equation (23).

	Coefficients ( *n*’ = *ln*(*n*))		
Sample Size	a	b	c
*n* < 50	0.0112 − 0.0012 *n’*	1.0076 − 0.0026 *n’*	− *ln*(*θ_a_*) − 0.2101 + 0.0107*n’*
50 ≤ *n* < 500	0.0131 − 0.0017 *n’*	1.0009 − 0.0016 *n’*	− *ln*(*θ_a_*) − 0.1980 + 0.0088*n’*
*n* ≥ 500	0.0069 − 0.0007 *n’*	0.9850 − 0.0008 *n’*	− *ln*(*θ_a_*) − 0.1581 + 0.0025*n’*

**Figure 3:**
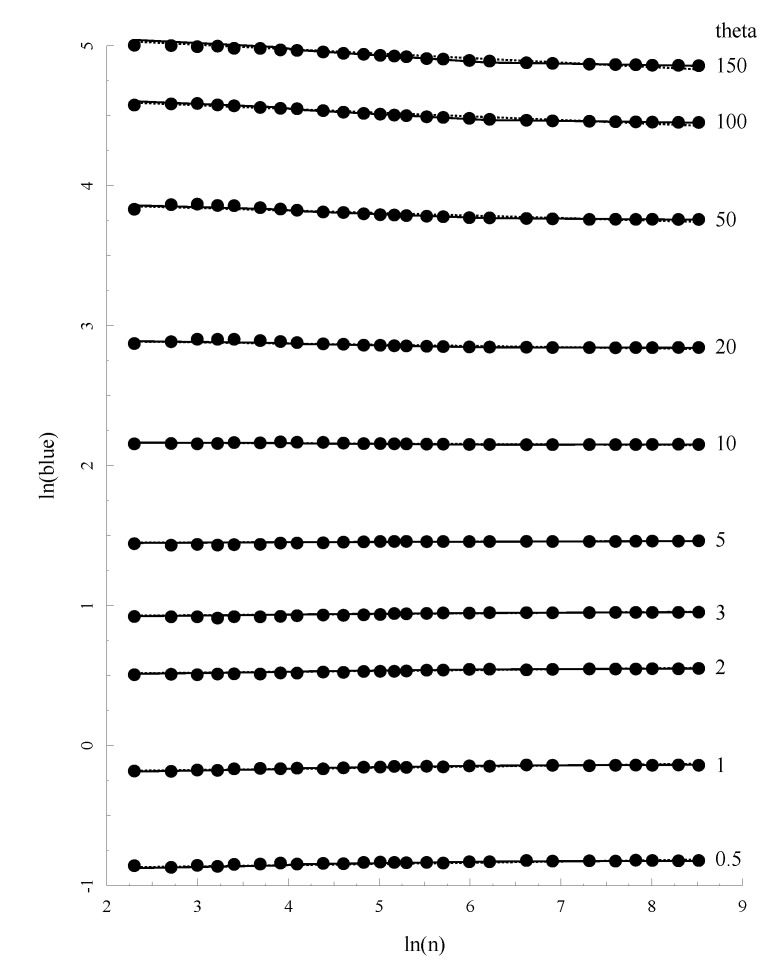
The relationship between Allelic Blue estimate *θ_a_*, *θ* and sample size. Solid lines represents the prediction of *θ_a_* based on Equation (23) with the coefficients given in Table 2

Figure 3 plots the relationship between *θ*, sample size (*n*) and the allelic BLUE estimates (*θ_ab_*) for a subset of these parameter combinations. It is easy to see that the match between prediction and the mean value of *θ_a_* is excellent. 

**Figure 4:**
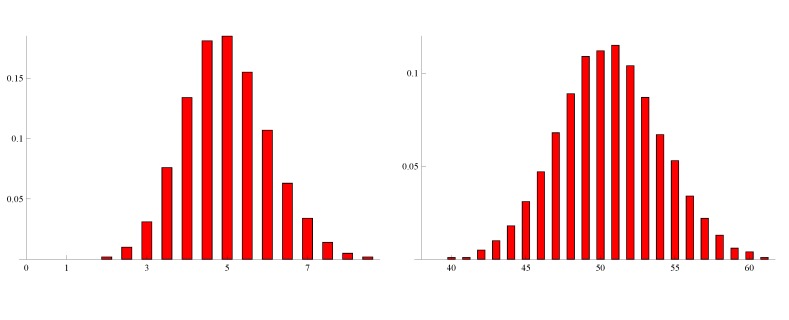
Distribution of ${\hat{\theta }}_{a}$ based on 1000 simulated samples. (**Left**) *n* = 500 and *θ* = 5; (**right**) *n* = 2000 and *θ*= 50.

Figure 4 shows the distributions of the estimate ${\hat{\theta }}_{a}$ from simulated samples in the case of *n* = 500 with *θ* = 5, and *n* = 2000 with *θ* = 50, respectively. It appears in both cases that the normalities are sufficiently accurate approximations, which is indeed expected from the theory of least squares estimators. 

The ultimate measure of the quality of an estimator is its bias and standard deviation for samples independent of those used to derive the estimator. Therefore, we simulated another set of samples for a number of combinations of *θ* and *n* and applied ${\hat{\theta }}_{a}$, as well as UPBLUE to these samples. Table 3 presents the summary of these simulations, particularly the efficiency of the new approach. 

**Table 3:** Performance of ${\hat{\theta }}_{a}$.

*θ*	*n*	mean ${\hat{\theta }}_{a}$	SE	*SD* *_min_*	Speed	Ratio
2	20	1.97	1.00	0.97	0.00	10
	50	1.98	0.84	0.81	0.00	59
	100	1.98	0.75	0.74	0.00	280
	500	2.00	0.63	0.61	0.02	1,569
	1,000	2.00	0.58	0.58	0.27	1,615
	2,500	2.00	0.54	0.54	2.59	3,104
5	20	4.93	1.90	1.83	0.00	4.5
	50	4.96	1.52	1.48	0.00	23
	100	4.99	1.34	1.30	0.00	78
	500	5.01	1.08	1.05	0.04	970
	1,000	5.00	1.00	0.98	0.47	1,306
	2,500	4.91	0.87	0.90	4.90	1,565
20	20	20.33	5.84	5.52	0.00	2.0
	50	20.27	4.20	4.05	0.01	4.4
	100	20.06	3.47	3.37	0.06	8.7
	500	20.04	2.56	2.49	0.18	359
	1,000	19.99	2.32	2.26	0.91	727
	2,500	19.96	2.07	2.04	16	842
50	20	50.92	12.58	12.51	0.01	1.6
	50	50.45	8.68	8.59	0.02	2.5
	100	50.09	6.99	6.79	0.07	3.8
	500	50.06	4.70	4.58	0.97	72
	1,000	49.90	4.15	4.06	3.6	206
	2,500	49.80	3.94	3.57	42	356
100	20	100.25	23.47	24.03	0.01	1.5
	50	100.20	15.52	15.87	0.15	1.7
	100	99.97	12.24	12.08	0.20	2
	500	100.38	7.86	7.48	4	22
	1,000	99.89	6.59	6.47	16	49
	2,500	99.69	5.58	5.54	75	202

Note: Speed is the average CPU time (in seconds) for obtaining ${\hat{\theta }}_{a}$ for a simulated sample in a Linux machine with a 2.3-Ghz CPU. *SDmin* is the minimum standard deviation computed as the square root of Equation (28) in [18]. Ratio is the ratio of speed for UPBLUE [13] and speed of ${\hat{\theta }}_{a}$.

Table 3 shows that the speed of ${\hat{\theta }}_{a}$ increases with the sample size slowly, while it increases faster with *θ*. This is because ${\hat{\theta }}_{a}$’s speed is dependent on the number of alleles in the sample, which is more closely related to *θ* than sample size. In comparison, UPBLUE is considerably less efficient, particularly with increasing sample size. Take the case of *θ* = 100 and *n* = 5000, it takes on average about one minute for ${\hat{\theta }}_{a}$ to complete the estimation, while it takes about 6 h for UPBLUE to do the same. 

## 3. Exploring *θ* in Data from the 1000 Genomes Project 

The 1000 Genomes Project generated a very valuable set of genome-wide polymorphism data [15] for which the newly developed Allelic Blue estimator is applicable. Phase I (May, 2012, release) contains polymorphism, as well as inferred phases compiled from 1092 individuals from 14 different populations. The rich information captured by the genome-wide polymorphism deserves to be analyzed from various angles [20], and our main purpose here is to illustrate that our efficient estimator of *θ* provides additional insight into the pattern of polymorphism in addition to the conventional estimates. We chose to report the analysis for a subset of samples, which consists of the three African samples (YRI, LWKand ASW) with 246 individuals (thus, a sample size of *n* = 492). 

In our analysis, each of the 22 autosomal chromosomes was divided into non-overlapping consecutive windows of 2000 bps (within which the average impact of recombination should be negligible), and *θ* was estimated for each window. Since the phases of SNPs for each individual were the result of inference, there are some segments in which the quality of inference appears to be poor due to an unreasonably larger number of inferred alleles than the number of SNPs. We thus removed all of the segments in which there is evidence of either recombination or a poor quality of inference, that is, when the number of alleles is larger than the number of SNPs plus one. It should be noted that the SNPs reported in the 1000 Genomes Project data set are those that passed various quality controls and filtering. In our analysis, no further filtering is applied, except for the aforementioned exclusion of segments that are suspected to be the result of poor phrase inference. A total of 648,903 windows were analyzed. This analysis required about 3 h to complete the estimates of *θ* in a desktop computer equipped with an Intel Xeon CPUs at 3.33 Ghz. In comparison, UBPLUE ran a couple days without being able to finish the same task. 

**Figure 5:**
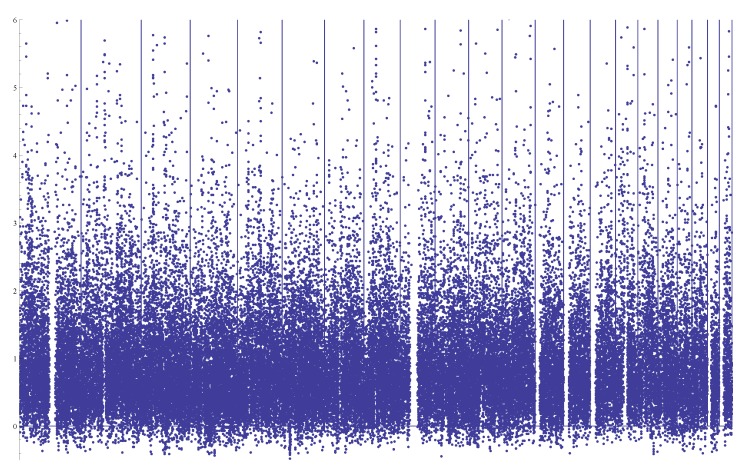
Plot of (${\hat{\theta }}_{W}$ ${\hat{\theta }}_{T}$)/${\hat{\theta }}_{T}$ along 22 autosomes with windows of a size of 2000 bps (each dot represents a mean over 10 consecutive windows). Chromosomes 1 to 22 are presented from left to right separated by vertical lines. The overall mean value is 0.96.

**Figure 6:**
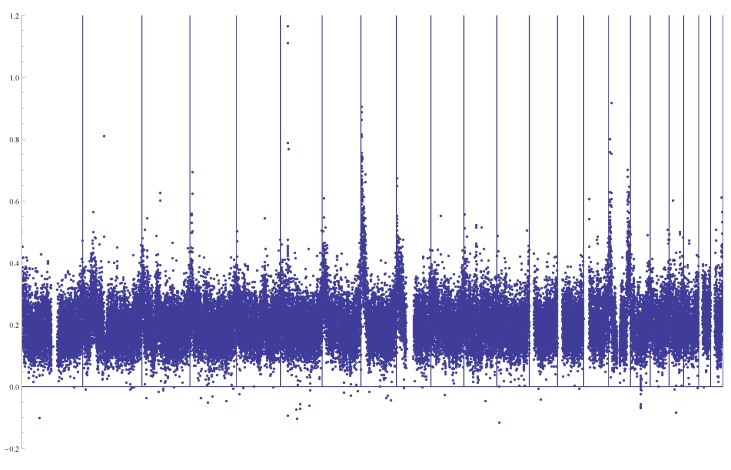
Plot of (${\hat{\theta }}_{a}$ − ${\hat{\theta }}_{W}$)/${\hat{\theta }}_{W}$ along 22 autosomes with windows of a size of 2000 bps (each dot represents a mean over 10 consecutive windows). Chromosomes 1 to 22 are presented from left to right separated by vertical lines. The overall mean value is 0.20.

Since both Watterson’s estimator ${\hat{\theta }}_{W}$ and Tajima’s estimator ${\hat{\theta }}_{T}$) are widely used, we present our results in terms of the relative values with regard to ${\hat{\theta }}_{W}$ and contrast them with the relative values of ${\hat{\theta }}_{W}$ to ${\hat{\theta }}_{T}$. Since testing neutrality is not the purpose, we do not employ testing statistics, such as Tajima’s test [19] or Fu and Li’s tests [7]. Figure 5 plots the proportional difference between ${\hat{\theta }}_{W}$ = *K*/*an* (*K* is the number of segregating sites and an is a constant depending on the sample size) and ${\hat{\theta }}_{T}$. The overwhelming characteristic of the plot is that *θ*_*W*_ is, in general, larger than ${\hat{\theta }}_{T}$, with an average of 1.96-times the value of ${\hat{\theta }}_{T}$; similar patterns were observed previously (for example, [20]). In general, an estimated *θ* can be viewed as a weighted average of SNPs of various sizes. ${\hat{\theta }}_{T}$ gives on average more weight to SNPs that occurred long ago than those arisen recently, while ${\hat{\theta }}_{W}$ gives equal weight to every SNP. Therefore, elevated ${\hat{\theta }}_{W}$ values across the whole genome compared to ${\hat{\theta }}_{T}$ were considered as evidence of recent population growth. It should be noted that there is no obvious extended regions with smaller or larger values for *θ*_*W *_ or *θ_T_*. In comparison, Figure 6 plots the proportional difference between ${\hat{\theta }}_{a}$ and ${\hat{\theta }}_{W}$. 

The overwhelming pattern shown in Figure 6 is again that ${\hat{\theta }}_{a}$ in general is larger than ${\hat{\theta }}_{W}$, which means that the difference to ${\hat{\theta }}_{T}$ will be more pronounced than that of ${\hat{\theta }}_{T}$. This is the result that more weight is given to recent mutations than the old ones in ${\hat{\theta }}_{a}$. Beside some sporadic large values, there are regions at either the beginning or the end of some chromosomes that yield considerably elevated values of ${\hat{\theta }}_{a}$ (for example, for chromosomes 7, 8, 9, 16 and 22). We are not sure how to interpret these patterns, but suspect that they may partially suggest the decreased quality of phase inference at the beginning and end of chromosomes. 

## 4. Discussion and Conclusions 

The Allelic BLUE estimator of *θ* presented in this paper is a high quality estimator with little bias and its variance nearly as small as the minimum achievable variance. Furthermore, it is highly efficient computationally, because its speed depends on the number of distinct alleles in a sample rather than the sample size. This later characteristic makes it very useful for estimating *θ* for large samples and for situations in which a large region (or the whole genome) is sequenced, while *θ* needs to be estimated for successive windows of a genome, such as the case of 1000 Genomes Projects. Since *θ_a_* and UPBLUE are both based on the same idea of utilizing phylogenetic information in a sample with generalized linear regression, their estimates are highly correlated, which are seen in both the simulation and in real data. However, since *θ_a_* is computationally much more efficient, it is thus superior to UPBLUE [13] and, thus, can replace UPBLUE for general use. The analysis of the polymorphism from the 1000 Genomes Project shows that although UPBLUE is a relatively efficient estimator among sophisticated estimators; it has nearly reached its limit for exploratory data analysis for large genome projects. Therefore, the new Allelic BLUE estimator arrival is timely. 

One additional utility of the new estimator ${\hat{\theta }}_{a}$ is for estimating *θ* from a collection of distinct alleles, which are collected without recording the multiplicity of each allele, as long as the number of alleles examined is roughly known. Such situations sometime arise when the collection of data is focused on identifying distinct alleles, such as in the survey of infectious pathogens or when data are collected over years and pooled from multiple sources. To illustrate this utility, we simulated samples of size 200 with *θ* = 10. If only the distinct alleles are recorded (which implicitly assumes that the sample size is the same as the number of distinct alleles), then *θa* yields an average value of 22.8, which is more than twice as large as the true value. However, if the sample size used in the estimation is 20% smaller or larger than the actual value (thus, 160 and 240, respectively), the corresponding ${\hat{\theta }}_{a}$ are 10.6 and 9.5, respectively, both of which are quite close to the true *θ* value. 

The Allelic BLUE estimator is developed under the assumption of one single random mating population evolving according to the Wright–Fisher model with a constant effective population size. The restriction to constant population size results in an estimator of average *θ* since the MRCAof a sample, which is comparable to some classical estimators and, thus, provides an informative contrast to other estimators (and may be used to construct hypothesis tests in the future). However, the restriction does make the method unsuitable for exploring historical changes in effective population sizes. On the other hand, the theoretical foundation for exploring changes in population sizes in the linear regression framework has been established in this paper, and we will study its statistical properties, as well as its application elsewhere. 

The Java programs for performing the Allelic Blue estimation can be downloaded from the author’s web page [21]. 
